# The Influence of Supervisor–Postgraduate Relationship on Master’s Students’ Research Learning Engagement—The Mediating Effect of Academic Aspiration

**DOI:** 10.3390/bs14040334

**Published:** 2024-04-16

**Authors:** Jianhe Zhang, Meiyin Wu, Guangjin Zhang

**Affiliations:** 1 Undergraduate Institute, China University of Geosciences (Wuhan), Wuhan 430074, China; jianhz@cug.edu.cn; 2 Institute of Education, China University of Geosciences (Wuhan), Wuhan 430074, China; 3 Public Administration Institute, China University of Geosciences (Wuhan), Wuhan 430074, China; zhangguangjin@cug.edu.cn

**Keywords:** master’s students, supervisor–postgraduate relationship, academic aspiration, research learning engagement

## Abstract

Research learning engagement is the basic element of master’s students’ innovation output, and the supervisor is the first responsible body for master’s students’ cultivation. Exploring the influence of the supervisor–postgraduate relationship on master’s students’ research learning engagement, with a focus on the mediating role of academic aspiration, is of great significance for the improvement of master’s students’ cultivation quality. We surveyed 569 master’s students at a university in Wuhan, China, using 3 measurement tools: the Supervisor–Postgraduate Relationship Scale, the Research Learning Engagement Scale, and the Academic Aspirations Scale. The results showed that: (1) The supervisor–postgraduate relationship positively and significantly predicted master’s students’ research learning engagement, and academic aspiration played a fully mediating role in the process. (2) There were differences in the effects of the three dimensions of the supervisor–postgraduate relationship on master’s students’ research learning engagement, with research collaboration having the greatest total effect on the impact of master’s students’ research learning engagement. This study emphasizes the importance of the supervisor–postgraduate relationship and academic aspirations and provides some implications for improving the research learning engagement of master’s students.

## 1. Introduction

As the highest level of national education, postgraduate education shoulders the important mission of high-level talent cultivation and innovation and is an important cornerstone of national development and social progress [1,2]. According to data on “The Basic Situation of National Education Development in 2022”, provided by China’s Ministry of Education in March 2023, there was approximately a total of 1,242,500 postgraduate students enrolled in China in 2022, an increase of 5.61% over the previous year. Among them, there were roughly 1,103,500 master’s students and 139,000 doctoral students enrolled. The reality of postgraduate education’s scale expansion indicates that China has joined the ranks of major countries and is striving to develop into a powerful country of postgraduate education, and the key to achieving the transformation lies in the enhancement of postgraduate students’ cultivation quality [3]. Postgraduate students and supervisors, who are the two main subjects of postgraduate education, are the two key elements affecting the quality of postgraduate cultivation in the micro-context of “cultivation quality” [4]. Particularly, as a significant indicator of postgraduate students’ research capacity and quality, research learning engagement is a facilitating factor in postgraduate students’ innovation output [5], which can ensure the effective improvement of postgraduate students’ cultivation quality [6]. As the first responsible body of postgraduate students’ cultivation, supervisors play a crucial role in improving the cultivation quality of postgraduate students [7]. In November 2020, the “Code of Conduct for Supervisors of Postgraduate Students”, issued by China’s Ministry of Education, explicitly stipulated that “building a harmonious teacher–student relationship” is one of the eight guidelines for the conduct of supervisors of postgraduate students. Specifically, “building a harmonious teacher–student relationship” requires supervisors to “implement the fundamental task of establishing moral education, strengthen humanistic care, pay attention to graduate students’ academic and employment pressure and psychological health, and establish a good teacher–student interaction mechanism. They should not insult the personality of postgraduate students or engage in inappropriate relationships with them”. As the most fundamental and essential educational relationship in the field of postgraduate education, the supervisor–postgraduate relationship has a direct impact on the improvement of postgraduate cultivation quality [8,9]. Generally, postgraduate education can be vertically divided into two levels: master’s education and doctoral education. In the background of the universal development of higher education, master’s education not only bears the responsibility of reserving students for China’s doctoral education, but also shoulders the mission of serving as a supply pool for the cultivation of high-level talents in China [10]. Therefore, this study discusses the relationship between the supervisor–postgraduate relationship and the research learning engagement of master’s students, with a particular focus on the question of “how” the supervisor–postgraduate relationship affects the research learning engagement of master’s students. This not only helps to discover some important laws in the field of master’s students’ education but can also provide a certain theoretical foundation and practical basis for the improvement of the quality of master’s cultivation in China.

## 2. Literature Review and Research Hypotheses

### 2.1. Supervisor–Postgraduate Relationship and Research Learning Engagement

According to Marx, “Man is the sum of all social relations” [11]. In the field of postgraduate education, the relationship between supervisors and postgraduates, as the most basic social relationship, mainly refers to a teaching relationship formed with postgraduate students to complete coursework, participate in research projects, and write dissertations under the guidance of supervisors, all in the process of learning how to conduct research [12]. In other words, this is essentially a complex relationship system with the linkage of knowledge inheritance and innovation, and the integration of various relationship types, including academic guidance, research collaboration, and emotional interaction [13].

At present, the academic community has not yet formed a consistent perception about the dimensional division of the supervisor–postgraduate relationship, but it basically covers the three aspects of academic guidance, research collaboration, and emotional interaction [14,15]. Among them, academic guidance refers to the practical guidance that supervisors provide to postgraduate students during their cultivation process. This includes assistance with topics such as research methods, drafting a thesis, nurturing abilities, and stimulating interest. Research collaboration means that in the cultivation process of postgraduate students, supervisors provide postgraduate students with good research conditions, sufficient opportunities to participate in the subject, and reasonable remuneration for research. Emotional interaction means that in the cultivation process of postgraduate students, supervisors provide postgraduate students with care, support, encouragement, respect, recognition, etc.

As an in-depth study of learning engagement at the research level, research learning engagement mainly refers to the time input, effort level, and learning intensity of postgraduate students in research, study, and academic activities [16]. In the academic community, the “cognitive–emotional–behavioral” three-dimensional structure of learning engagement is now widely acknowledged [17]. In this study, research learning engagement includes cognitive engagement, emotional engagement, and behavioral engagement in three dimensions, which not only reflect the state of individual participation in cognitive strategies, such as critical thinking, positive emotion, and identification of research and learning on the implicit level, but also reflect individual behavior proactivity on the explicit level [18,19].

According to Mills’ significant others theory, individuals are more likely to have a higher sense of self-worth when they receive recognition and support from significant others [20]. On the other hand, Rohner’s interpersonal acceptance–rejection theory suggests that individuals may experience psychological and behavioral problems when there is a lack of trust and support from significant others [21]. Studies conducted both nationally and internationally have revealed that students’ interactions with significant others have an impact on their development [22,23,24]. A national cross-sectional study from the United States showed that supervisors, as the first person responsible for the cultivation of postgraduate students, acted as significant others for postgraduate students during their academic years in the vast majority of cases and played a very important role in the development of postgraduate students’ careers in terms of intellectual guidance, emotional support, and social resources [25]. There have been a series of empirical explorations on the effect of the supervisor–postgraduate relationship on postgraduate students’ research learning engagement. Based on a study by psychology graduate students at Midwestern University, Cronan-Hillix et al. demonstrated that mentoring support can positively and significantly predict postgraduate students’ time engagement in research [26]. American scholars Deci et al. also found that individuals will devote more energy to research activities with the support of their supervisor [27]. On the other hand, based on a study of postgraduate students at the University of Queensland, UK, McEvoy et al. noted that the emotional support of supervisors had a significant impact on postgraduate students’ engagement in academic research [28]. Based on the data analysis of the “Satisfaction Survey of China Postgraduate Students”, Chinese scholars Li et al. discovered that the level of supervisors’ guidance had a positive and significant direct effect on master’s students’ research engagement [29]. Chinese scholars Ma et al. (2023) also confirmed that faculty support (affective interactions) positively predicted graduate students’ engagement in research [30]. On this basis, the following hypotheses were put forward for this study:

**H_1_:**  *The supervisor–postgraduate relationship positively and significantly influences research learning engagement of master’s students.*

**H_1a_:**  *Academic guidance positively and significantly influences master’s students’ research learning engagement.*

**H_1b_:**  *Research collaboration positively and significantly influences master’s students’ research learning engagement.*

**H_1c_:**  *Emotional communication positively and significantly influences master’s students’ research learning engagement.*

### 2.2. The Mediating Effect of Academic Aspiration

External driving factors frequently work through internal driving factors, so it is necessary to examine the influence path of the supervisor–postgraduate relationship on master’s students’ research learning engagement.

According to Bandura’s ternary interaction theory, it is known that individual behavior is generally influenced by a combination of individual and environmental factors [31], which implies that master’s students’ engagement in research learning is not only influenced by the supervisor–postgraduate relationship regarding the environmental factors, but also the individual factors. Academic aspiration may be one of the intrinsic traits of master’s students that may have a significant impact on their engagement in research learning. Academic aspiration is an individualized and organic combination of aspiration and interest, internal motivation, and external motivation, which mainly refers to an academic aspiration for a particular major or subject area arising from an assessment of an individual’s interest and potential, which is specifically manifested as a tendency to have a research interest, to identify with academic value, and to aspire to engage in academic work [32]. The basic connotation of academic aspiration has been interpreted by scholars both domestically and internationally. Some of these interpretations are divided into four dimensions: academic enthusiasm, future confidence, career ambition, and self-expression [33], and others are summarized based on four aspects: academic enthusiasm, academic resilience, academic identity and professional commitment, and academic ambition [34].

It has been shown that there is a mutually reinforcing relationship between academic ambition, as a support for postgraduate students to invest a certain amount of time and energy in research, and learning engagement [35]. Some scholars have confirmed and further pointed out that postgraduate students’ interest in research can motivate them to increase their participation in research activities [36], and research engagement can also have a significant positive impact on their academic aspiration [37]. Additionally, it was demonstrated by Mageau et al. that passion is linked to higher levels of participation in activities [38]. Therefore, this study suggests that academic aspiration may have an impact on postgraduate students’ research learning engagement. Based on Cohen’s social support theory, it is known that social support can have a generally gainful effect on individuals, and good social support can bring positive emotional experiences to individuals [39]. Ruiz-Alfonso et al. also argued that supportive environments and positive relationships are the most important influences for individuals to develop and maintain passion [40]. Within the field of postgraduate education, supervisors’ support is one of the important social supports that master’s students can perceive, which may play a key role in stimulating their academic aspirations and motivating their research motivation. It has been shown that the supervisor–postgraduate relationship is an important factor affecting the academic aspirations of postgraduate students, and a positive supervisor–postgraduate relationship is conducive to enhancing postgraduate students’ academic aspirations [32]. In particular, affective interaction between supervisors and master’s students positively predicted academic commitment, and the higher the academic interactions between supervisors and master’s students, the higher the academic aspirations of master’s students [41]. On the other hand, the study by Li et al. demonstrated that postgraduate students’ academic enthusiasm would likely increase with the intensity of the supervisor in terms of academic guidance and life care, and that there would also likely be a significant increase in the amount of time for independent study and scientific research activities [42]. In addition, other studies have confirmed that academic aspiration is an intrinsic motivation for postgraduate students to engage in research activities, and that mentoring can promote the growth of postgraduate students’ research ability by stimulating their academic aspiration [43]. This may imply that master’s students with a more satisfactory perception of the supervisor–postgraduate relationship have higher academic aspirations, which in turn results in increased engagement in research learning, and finally, improvements in their research ability. On this basis, the following hypotheses were put forward for this study:

**H_2_:**  *The supervisor–postgraduate relationship positively and significantly affects the academic aspirations of master’s students.*

**H_2a_:**  *Academic guidance positively and significantly affects the academic aspirations of master’s students.*

**H_2b_:**  *Research collaboration positively and significantly affects the academic aspirations of master’s students.*

**H_2c_:**  *Affective interaction positively and significantly affects the academic aspirations of master’s students.*

**H_3_:**  *Academic aspiration positively and significantly affects master’s students’ research learning engagement.*

**H_4_:**  *Academic aspiration has a mediating effect between the supervisor–postgraduate relationship and the master’s students’ research learning engagement.*

**H_4a_:**  *Academic aspiration has a mediating effect between academic guidance and master’s students’ research learning engagement.*

**H_4b_:**  *Academic aspiration has a mediating effect between research collaboration and master’s students’ research learning engagement.*

**H_4c_:**  *Academic aspiration has a mediating effect between affective interaction and master’s students’ research learning engagement.*

In summary, scholars at home and abroad have discussed the relationship between the supervisor–postgraduate relationship, academic aspiration, and research learning engagement. Most scholars have paid attention to the influence of the external environmental factors of the supervisor–postgraduate relationship on individual psychology and behavior in the field of postgraduate education, and more scholars have begun to pay attention to the relevant research on academic aspiration and research learning engagement, but there are still gaps in the existing research. Firstly, existing studies have provided sufficient theoretical empirical summaries of the connotation and nature of the supervisor–postgraduate relationship [12,13], but most scholars have focused on relevant empirical explorations from one or some sub-dimensions of the supervisor–postgraduate relationship [26,27,28,29], lacking a relatively comprehensive empirical test of the supervisor–postgraduate relationship. Secondly, existing studies mainly focus on undergraduates and doctoral students in the stage of higher education [44,45], and there is a gap in the studies concerning master’s students, in which the supervisor–postgraduate relationship has an impact on research learning engagement through academic aspiration.

**Figure 1:**
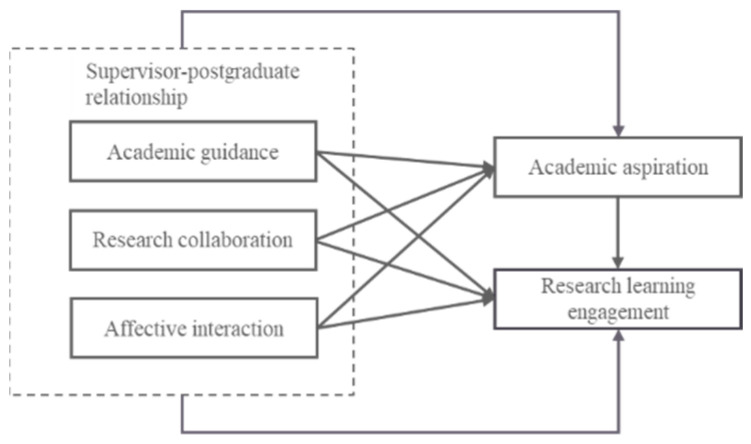
Hypothesis model of the mediating effect of academic aspiration.

Therefore, based on Bandura’s ternary interaction theory and the results of existing empirical explorations, in this study, we constructed a mediation effect model to explore the relationship between the supervisor–postgraduate relationship and master’s students’ research learning engagement and investigate the role of academic aspiration in the process. Then, we explained master’s students’ research learning engagement behaviors in terms of a combination of internal and external factors, to provide empirical evidence and useful references for the high-quality cultivation of master’s students. The relationship between the variables is shown in Figure 1.

## 3. Research Design

### 3.1. Participants

Due to the COVID-19 pandemic and other objective factors, this study mainly used the random sampling method to distribute the questionnaires through a combination of online and offline methods. The electronic questionnaires were distributed online through social media (WeChat, QQ, etc., in China), and the paper questionnaires were distributed offline in seminar rooms, libraries, and so on. The questionnaire survey was conducted in October–November 2022, with master’s students from a university in Wuhan, China, as the study population. A total of 631 questionnaires were distributed in this survey, and after excluding invalid questionnaires, such as those with a response time of less than 120 s and wrong answers to polygraph questions (e.g., “This question is a lie detector question, please select ‘very inconsistent’; otherwise, this questionnaire is invalid”), 569 valid questionnaires (388 electronic questionnaires and 181 paper questionnaires) were recovered, with a validity rate of 90.2%.

Among them, in terms of gender distribution, there were 263 male students, accounting for 46.2%, and 306 female students, accounting for 53.8%. In terms of grade distribution, there were 160 first-year master’s students, accounting for 28.1%, 216 second-year master’s students, accounting for 38.0%, and 193 third-year master’s students, accounting for 33.9%. In terms of the distribution of majors, there were 277 students in humanities and social sciences, accounting for 48.7%, and 292 students in science and technology, accounting for 51.3%. In terms of the willingness to study for a doctoral degree, 133 master’s students planned to study for a doctoral degree, accounting for 23.4%, and 436 did not plan to study for a doctoral degree, accounting for 76.6%.

### 3.2. Measures

#### 3.2.1. Supervisor–Postgraduate Relationship Scale

The Supervisor–Postgraduate Relationship Scale was adapted from a related study by Noe [46], and consists of 17 question items, such as “My supervisor always gives me prompt feedback on my problems”. The scale was scored on a 5-point Likert scale, with higher scores indicating a better supervisor–postgraduate relationship, as perceived by master’s students. The data of the scale were randomly divided into two homogeneous samples, and in the empirical test of sample 1 (a total of 293), the Cronbach’s α coefficient, the KMO value, and the Bartlett sphericity test value of the scale were 0.964 > 0.7, 0.959, and 4855.928, *p* < 0.001. Exploratory factor analysis extracted the total of three common factors, named academic guidance, research collaboration, and affective interaction, which explained 77.8% of the variation. In the empirical test of sample 2 (a total of 276), the results of the confirmatory factor analysis revealed that the structural validity was ideal, with the *χ*^2^/*df* being 2.749 < 5, the RMSEA being 0.079 < 0.8, the SRMR being 0.034 < 0.8, the NFI being 0.938 > 0.9, the CFI being 0.959 > 0.9, the TLI being 0.952 > 0.9, and the IFI being 0.959 > 0.9. The factor loadings of each item took the values of 0.692 to 0.938 > 0.5, the AVE took the values of 0.703 to 0.756 > 0.5, and the CR took the values of 0.904 to 0.953 > 0.7, which meant that the convergent validity was ideal. The fitting indexes of the three-factor model were better than those of other nested models, which meant the discriminative validity was ideal. The results showed that the reliability and validity of the Supervisor–Postgraduate Relationship Scale were good.

#### 3.2.2. Research Learning Engagement Scale

The Research Learning Engagement Scale was adapted from related studies by Fredricks et al. [18] and Yuan [19], and consists of 17 question items, such as “Exploring academic issues can bring me pleasure”. The scale was scored on a 5-point Likert scale, with higher scores indicating higher research learning engagement of master’s students. In the empirical test of sample 1, the Cronbach’s α coefficient, the KMO value, and the Bartlett sphericity test value of the scale were 0.953 > 0.7, 0.950, and 4389.071, *p* < 0.001. Exploratory factor analysis extracted the total of three common factors, named cognitive engagement, affective engagement, and behavioral engagement, which explained 80.0% of the variation. In the empirical test of sample 2, the results of the confirmatory factor analysis revealed that the structural validity was ideal, with the *χ*^2^/*df* being 2.274 < 5, the RMSEA being 0.068 < 0.8, the SRMR being 0.038 < 0.8, the NFI being 0.916 > 0.9, the CFI being 0.951 > 0.9, the TLI being 0.944 > 0.9, and the IFI being 0.951 > 0.9. The factor loadings of each item took the values of 0.636 to 0.892 > 0.5, the AVE took the values of 0.588 to 0.674 > 0.5, and the CR took the values of 0.909 to 0.922 > 0.7, which meant the convergent validity was ideal. The fitting indexes of the three-factor model were better than those of other nested models, which meant the discriminative validity was ideal. The results showed that the reliability and validity of the Research Learning Engagement Scale were good.

#### 3.2.3. Academic Aspiration Scale

The Academic Aspiration Scale was adapted from a related study by Liu et al. [32], and consists of 7 question items, such as “I am interested in my major and related fields”. The scale was scored on a 5-point Likert scale, with higher scores indicating higher academic aspiration of master’s students. In the empirical test of sample 1, the Cronbach’s α coefficient, the KMO value, and the Bartlett sphericity test value of the academic aspiration scale were 0.900 > 0.7, 0.874, and 1254.227, *p* < 0.001. Only 1 common factor was extracted by exploratory factor analysis, explaining 63.2% of the variation. In the empirical test of sample 2, the results of confirmatory factor analysis showed that the structural validity was ideal, with the *χ*^2^/*df* being 2.705 < 5, the RMSEA being 0.079 < 0.8, the SRMR being 0.033 < 0.8, the NFI being 0.970 > 0.9, the CFI being 0.981 > 0.9, the TLI being 0.966 > 0.9, and the IFI being 0.981 > 0.9. The factor loadings of each item took the values of 0.639 to 0.798 > 0.5, the AVE was 0.542 > 0.5, and the CR was 0.892 > 0.7, which meant the convergent validity was ideal. Since the Academic Aspirations Scale is a unidimensional scale, there is no measure of discriminant validity. The results showed that the reliability and validity of the Academic Aspiration Scale were good.

### 3.3. Data Analysis

Firstly, SPSS 26.0 and Amos 24.0 software were used for reliability and validity tests, as well as common method bias tests. Secondly, SPSS 26.0 software was used for descriptive statistical analysis, correlation analysis, and difference analysis. Finally, Amos 24.0 software was used to construct a structural equation model to test the mediating effect of academic aspiration in the supervisor–postgraduate relationship and its three dimensions with research learning engagement.

### 3.4. Common Method Bias Test

In this study, all questionnaires were answered anonymously. Combined with existing research suggestions [47], exploratory factor analysis and confirmatory factor analysis were conducted on the data to test whether there was serious common method bias. In the empirical test of sample 1, the results of exploratory factor analysis showed that a total of 7 common factors with eigenvalues greater than 1 were extracted without rotation, with the first common factor explaining 46.8% of the variance, which was less than the judgmental criterion of 50.0% recommended by Hair et al. [48]. In the empirical test of sample 2, the results of confirmatory factor analysis of the single factor showed that the fit of the one-factor model was poorly fitted, with a *χ*^2^/*df* of 5.635, an RMSEA of 0.130, an SRMR of 0.114, an NFI of 0.585, a CFI of 0.630, a TLI of 0.611, and an IFI of 0.631. In summary, there were no serious common methodological biases in this study.

## 4. Results

### 4.1. Descriptive Statistics and Correlation Analysis of Each Variable

The results of the statistical analysis of the mean, standard deviation, and correlation coefficient of each variable are detailed in Table 1. From the data in the table, it can be seen that there was a two-by-two positive correlation between the supervisor–postgraduate relationship and its three dimensions, academic aspiration, and research learning engagement, and all of them were significant at the level of 0.01, indicating that the data in this study can be analyzed subsequently.

### 4.2. Analysis of the Mediating Effect of Academic Aspiration

The structural equation was used to test the mediating effect of academic aspiration between the supervisor–postgraduate relationship and master’s students’ research learning engagement. Because of the large number of measurement items included in the three scales and the small sample size of this study, modeling directly using the original questions is prone to random error [49]. Therefore, as recommended by Wu et al. (2011) [49], the three scales were first packaged using the factorial method before being included in the structural equation analysis in order to avoid random errors. Specifically, the internal consistency method was used to package the two multidimensional scales of the supervisor–postgraduate relationship and research learning engagement into three indicators each, and the high and high loadings method of the factorial method was used to package the unidimensional scale of academic aspirations into two indicators.

**Figure 2:**
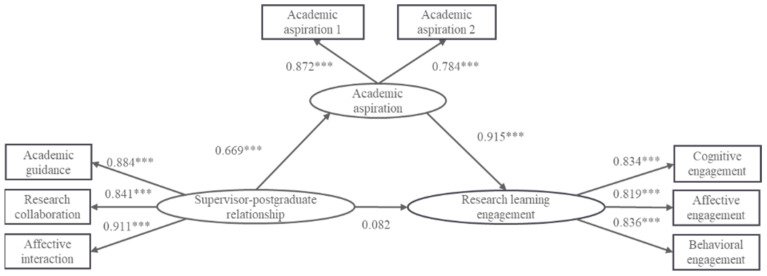
Model of the mediating effect of academic aspiration between the supervisor–postgraduate relationship and research learning engagement (M1). Note: *** *p* < 0.001.

First, the total effect of the supervisor–postgraduate relationship on the research learning engagement of master’s students was examined, and the results showed that the model fit well (*χ*^2^*/df* = 2.162, RMSEA = 0.045, SRMR = 0.024, GFI = 0.990, IFI = 0.996, TLI = 0.992, and CFI = 0.996). The supervisor–postgraduate relationship significantly positively predicted master’s students’ research learning engagement (*β* = 0.687, *p* < 0.001), which indicated that research hypothesis H_1_ was valid. Secondly, academic aspiration was added as a mediator variable to construct the intermediary effect model M1 (see Figure 2), and the results showed that the model fit was acceptable (*χ*^2^/*df* = 3.168, RMSEA = 0.062, SRMR = 0.022, GFI = 0.976, IFI = 0.989, TLI = 0.982, and CFI = 0.989). The supervisor–postgraduate relationship significantly positively predicted academic aspiration (*β* = 0.669, *p* < 0.001), academic aspiration significantly positively predicted research learning engagement (*β* = 0.915, *p* < 0.001), and the direct predictive effect of the supervisor–postgraduate relationship on research learning engagement was not significant (*β* = 0.082, *p* > 0.05), which indicated that research hypotheses H_2_ and H_3_ were valid.

The significance of the mediating effect was further tested by the bootstrap method of bias correction. If the 95% confidence interval (5000 repeated samples) does not include 0, it indicates that the mediating effect is significant. The results showed that (see Table 2) the total effect of the supervisor–postgraduate relationship on research learning engagement was significant, with a 95% confidence interval of [0.392, 0.574], and the mediating effect of the supervisor–postgraduate relationship on research learning engagement through academic aspiration was significant, with a 95% confidence interval of [0.335, 0.542], but the direct effect of the supervisor–postgraduate relationship on research learning engagement was no longer significant, with a 95% confidence interval of [−0.014, 0.128]. Therefore, academic aspiration played a fully mediating role between the supervisor–postgraduate relationship and research learning engagement, with a mediating effect value of 0.423 and an effect proportion of 88.12%, which indicated that research hypothesis H_4_ was valid.

**Figure 3:**
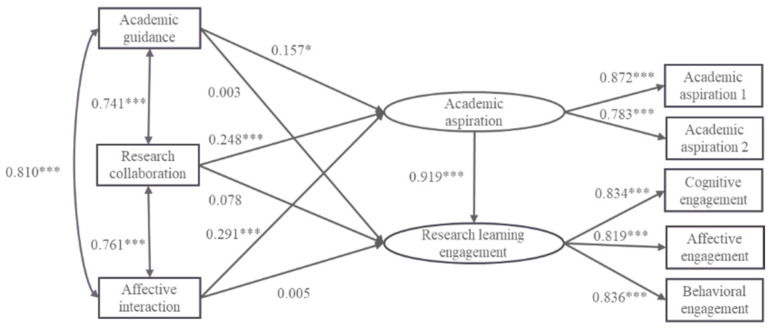
Model of the mediating effect of academic aspiration between the dimensions of supervisor–postgraduate relationship and research learning engagement (M2). Note: * *p* < 0.05, *** *p* < 0.001.

Academic guidance, research collaboration, and affective interaction were used as predictor variables, research learning engagement was used as an outcome variable, and academic aspiration was used as a mediator variable in order to measure the effect of three dimensions of the supervisor–postgraduate relationship on master’s students’ research learning engagement. Firstly, the total effect of the three dimensions of the supervisor–postgraduate relationship on master’s students’ research learning engagement was examined, and the results showed that the model fit well (*χ*^2^/*df* = 1.773, RMSEA = 0.037, SRMR = 0.019, GFI = 0.994, IFI = 0.998, TLI = 0.995, and CFI = 0.998). Academic guidance, research collaboration, and affective interaction all significantly and positively predicted master’s students’ research learning engagement (*β* = 0.147, *p* < 0.05; *β* = 0.305, *p* < 0.001; *β* = 0.268, *p* < 0.001), which indicated that research hypotheses H_1a_, H_1b_, and H_1c_ were valid. Secondly, academic aspiration was added as a mediating variable to construct the mediation effect model M2 (see Figure 3), and the results showed that the model fit was acceptable (*χ*^2^*/df* = 3.613, RMSEA = 0.068, SRMR = 0.018, GFI = 0.979, IFI = 0.990, TLI = 0.978, and CFI = 0.990). Academic guidance, research collaboration, and affective interaction all significantly and positively predicted academic aspiration (*β* = 0.157, *p* < 0.05; *β* = 0.248, *p* < 0.001; *β* = 0.291, *p* < 0.001), and academic aspiration significantly positively predicted research learning engagement (*β* = 0.919, *p* < 0.001), but the direct predictive effects of academic guidance, research collaboration, and affective interaction on research learning engagement were not significant (*β* = 0.003, *p* > 0.05; *β* = 0.078, *p* > 0.05; *β* = 0.005, *p* > 0.05), which indicated that research hypotheses H_2a_, H_2b_, and H_2c_ were all valid.

The significance of the mediating effect was further tested by the bootstrap method of bias correction. The results showed (see Table 3) that there were differences in the effects of the three sub-dimensions of the supervisor–postgraduate relationship on master’s students’ research learning engagement. Among them, academic aspiration played a full mediating role between research collaboration and research learning engagement, as well as between affective interaction and research learning engagement, while the mediating effect between academic guidance and research learning engagement was not significant, which indicated that research hypotheses H_4b_ and H_4c_ were valid, but research hypothesis H_4a_ was not valid.

## 5. Discussion

Based on the ternary interaction theory, this study constructed a mediation effect model of “supervisor–postgraduate relationship → academic aspiration → research learning engagement”, discussed the relationship between the supervisor–postgraduate relationship and master’s students’ research learning engagement, and focused on answering the question of “how” the supervisor–postgraduate relationship affects master’s students’ research learning engagement. To a certain extent, the research results can make an effective supplement to the studies related to the research learning engagement of master’s students, and at the same time provide some references for improving the quality of master’s students’ cultivation.

### 5.1. Supervisor–Postgraduate Relationship Positively and Significantly Predicted Master’s Students’ Research Learning Engagement

The results showed that there was a significant positive correlation between both the supervisor–postgraduate relationship and its three dimensions and master’s students’ research learning engagement, while both the supervisor–postgraduate relationship and its three dimensions had a significant positive prediction effect on master’s students’ research learning engagement. That is, the better the supervisor–postgraduate relationship was perceived by master’s students, the more they engaged in research learning, which was in line with the viewpoints of Mills’ significant others theory, and empirically extends the study of the supervisor–postgraduate relationship on learning engagement from undergraduate and doctoral fields to the master’s field. The results of the study also showed that the overall satisfaction with the supervisor–postgraduate relationship, as perceived by master’s students, was at a moderately high level, with the affective interaction dimension scoring the highest, followed by the academic guidance dimension, and the last dimension was the research collaboration dimension. Meanwhile, research collaboration had the greatest total effect on master’s students’ research learning engagement, while the affective interaction dimension was more important than the academic guidance dimension. This corresponds, to some extent, with the findings of Yuan [19], showing that the positive influence effect of mentoring on master’s students’ engagement in research and learning was relatively weak compared to supervisor autonomy and emotional support.

Based on Blau’s social exchange theory, it can be seen that all social activities of human beings are essentially an exchange, and the development and maintenance of interpersonal relationships are based on the mutual exchange of valuable resources among individuals, which can be not only economic resources, such as salary and welfare, but also social resources, such as respect and recognition [50]. To some extent, the supervisor–postgraduate relationship also contains the idea of exchange. The supervisor is an important person during the study period of master’s students, and when master’s students perceive that the supervisor provides them with opportunities for research collaboration and gives them a reasonable reward for participating in projects, they are more likely to increase their time and energy engagement in research and learning, and actively complete various tasks of research and learning. Similarly, when master’s students perceive the support, respect, and recognition from their supervisor, they are also more likely to reciprocate their supervisor’s expectations with “more engagement”, in order to develop and maintain a harmonious supervisor–postgraduate relationship.

Therefore, in order to ensure the quality of master’s students’ cultivation, it is crucial to build a high-quality supervisor–postgraduate relationship and to effectively enhance research learning engagement. According to material dialectics, it is necessary to adhere to the organic unity of “two-point theory” and “key point theory”, and to seize the main contradiction and the main aspects of the contradiction based on a comprehensive and balanced approach. This means that the construction of a high-quality supervisor–postgraduate relationship should start from the three dimensions of academic guidance, research collaboration, and affective interaction, focusing on optimizing the level of research collaboration between the supervisor and the postgraduate so as to effectively enhance the degree of master’s students’ research learning engagement.

### 5.2. Academic Aspiration Had a Fully Mediating Effect between the Supervisor–Postgraduate Relationship and Master’s Students’ Research Learning Engagement

The results showed that academic aspiration had a fully mediating effect on the influence of the supervisor–postgraduate relationship on master’s students’ research learning engagement. This meant that the direct effect of the supervisor–postgraduate relationship on master’s students’ research learning engagement was no longer significant after the introduction of academic aspiration as a mediating variable into the theoretical model, but rather, it had an indirect effect on master’s students’ research learning engagement through the mediating effect of academic aspiration. To a certain extent, this is consistent with the prediction of Bandura’s ternary interaction theory; that is, master’s students’ research learning engagement is the result of the joint action of environmental factors and individual factors, and further clarifies that if we want to motivate master’s students to increase their time investment, effort level, and learning intensity in research through the external environmental factor of the supervisor–postgraduate relationship, we need to stimulate the academic interest and ambition of master’s students in their specialisms and disciplinary fields of study. Weiner’s [51] attribution theory also points out that the factors affecting individual behavior mainly include internal and external factors, in which external factors tend to act on the internal factors to have an impact on individual behavior.

The results also showed that in the relationship between the three dimensions of the supervisor–postgraduate relationship and master’s students’ research learning engagement, the direct effect of research collaboration and affective interaction on master’s students’ research learning engagement was no longer significant after the introduction of academic aspiration as a mediating variable into the theoretical model. Both of them were indirectly affected through the mediator effect of academic aspiration on master’s students’ research learning engagement, whereas the mediating effect of academic aspiration in the relationship between academic guidance and master’s students’ research learning engagement was not significant. This meant that the effects of research collaboration and affective interaction on master’s students’ research learning engagement can be better exerted through academic aspiration. This is corroborated by Kahn’s [52] findings, showing that high-level research training can improve postgraduate students’ research engagement by enhancing their academic aspirations.

In summary, it can be seen that academic aspiration is an important internal driving force for master’s students to engage in research and learning, and it can strengthen the positive impact of the supervisor–postgraduate relationship on master’s students’ research learning engagement. In particular, it can strengthen the facilitating effect of research collaboration and affective interaction on master’s students’ research learning engagement. Meanwhile, compared to the supervisor–postgraduate relationship, which is an external environmental factor, the level of academic aspiration of master’s students scored the lowest overall, yet it had a stronger positive impact on master’s students’ research learning engagement. As a result, it is of positive significance in promoting the research learning engagement of master’s students to cultivate master’s students’ academic aspirations and fully stimulate their internal driving force of research and learning. The four-stage model of interest development points out that the formation and development of individual interest requires not only sufficient external support to stimulate and maintain situational interest, but also the continuous acquisition of positive emotional experience to form stable individual interest [53]. Thus, how can we cultivate the academic aspirations of master’s students? First, the cultivation units should create a positive research atmosphere to create favorable conditions for the nurturing of master’s students’ academic aspirations. For example, master’s students can be provided with opportunities to get in touch with new things and broaden their academic horizons through measures such as opening up channels for accessing academic resources, building a platform for sharing information on academic conferences, organizing high-level academic forums, encouraging international intercollegiate academic exchanges, and regularly organizing seminars for faculty members and postgraduate students in their disciplinary fields. Second, master’s students should be supported in their positive emotional experiences by their supervisors, who act as significant people during their enrollment. For example, supervisors should provide effective guidance and timely feedback to master’s students when they encounter problems, provide opportunities to participate in scientific research projects according to the needs of master’s students, as much as possible, allow master’s students to carry out research on their own within a certain range of topics, as well as provide care, encouragement, respect, and understanding of master’s students in their daily lives, etc., so as to enable master’s students to perceive positive emotional experiences from the three aspects of academic guidance, research collaboration, and affective interactions.

### 5.3. Limitations and Future Directions

This study clarified the influence of the supervisor–postgraduate relationship on master’s students’ research learning engagement, explored the mediating effect of academic aspiration, and provided a new perspective for improving the quality of master’s students’ cultivation, but at the same time, there are still some shortcomings that need to be improved. First, the research conclusions may only be applicable to China. This study only investigated Chinese master’s students, and there are some differences in master’s education around the world, so the conclusions of the study may not be generalizable globally. Second, the research methodology needs to be enriched. In this study, data were collected by self-reported questionnaires of master’s students, and all the data were cross-sectional, which was not enough to accurately reveal the causal relationship between the supervisor–postgraduate relationship and master’s students’ research learning engagement. Future studies can collect data from multiple time points and add questionnaires or interviews from the supervisors’ perspective to increase the persuasiveness of the findings. Third, the content of the study needs to be improved. This study explored the role of academic aspiration in the relationship between the supervisor–postgraduate relationship and master’s students’ research learning engagement, without considering the influence of other variables. In the future, more variables can be explored theoretically to enrich the research model so as to further explore the relationship between the supervisor–postgraduate relationship and master’s students’ research learning engagement, and to explore more effective methods for high-quality cultivation of master’s students.

## Figures and Tables

**Table 1:** Descriptive statistics and correlation analysis results of each variable (*n* = 569).

Variables	1	2	3	4	5	6
1. Supervisor–postgraduate relationship	1					
2. Academic guidance	0.944 **	1				
3. Research collaboration	0.879 **	0.741 **	1			
4. Affective interaction	0.932 **	0.810 **	0.761 **	1		
5. Academic aspiration	0.570 **	0.511 **	0.531 **	0.541 **	1	
6. Research learning engagement	0.617 **	0.554 **	0.580 **	0.582 **	0.818 **	1
*M*	3.721	3.751	3.572	3.785	3.493	3.633
SD	0.792	0.868	0.904	0.813	0.700	0.613

Note: ** *p* < 0.01

**Table 2:** Results of the path test of the intermediary effect model M1 (*n* = 569).

Effect	Influence Path	Effect Size	Boot SE	95% CI		Proportion of Effects
				LLCI	ULCI	
Total	Supervisor–postgraduate relationship → Research learning engagement	0.480	0.046	0.392	0.574	
Direct	Supervisor–postgraduate relationship → Research learning engagement	0.057	0.035	0.014	0.128	11.88%
Indirect	Supervisor–postgraduate relationship → Academic aspiration → Research learning engagement	0.423	0.052	0.335	0.542	88.12%

Note: Self-sampling results were based on 5000 self-sampling samples.

**Table 3:** Results of the path test of the mediated effect model M2 (*n* = 569).

Effects	Influence Path	Effect Size	Boot SE	95% CI		Proportion of Effects
				LLCI	ULCI	
Direct	Academic guidance → Research learning engagement	0.002	0.031	0.064	0.061	2.27%
Indirect	Academic guidance → Academic aspiration → Research learning engagement	0.086	0.048	0.002	0.186	97.73%
Direct	Research collaboration →Research learning engagement	0.044	0.027	0.008	0.096	25.43%
Indirect	Research collaboration → Academic aspiration → Research learning engagement	0.129	0.036	0.061	0.203	74.57%
Direct	Affective interaction → Research learning engagement	0.003	0.037	0.069	0.077	1.74%
Indirect	Affective interaction → Academic aspiration → Research learning engagement	0.169	0.053	0.065	0.273	98.26%

Note: Self-sampling results were based on 5000 self-sampling samples.

## Data Availability

The original contributions presented in this study are included in this article. Further inquiries can be directed to the corresponding author.
